# Supplementary material for: Quantum octets in high mobility pentagonal two-dimensional PdSe2
Source: Nat Commun. 2024 Jan 26;15:761. doi: 10.1038/s41467-024-44972-2 (PMC10817936; doi:10.1038/s41467-024-44972-2)
Supplement: Supplementary file 1 — Supplementary Information [file 41467_2024_44972_MOESM1_ESM.pdf]

Supplementary Information

Quantum Octets in High Mobility Pentagonal Two-Dimensional $PdSe_2$

Yuxin Zhang[1], Haidong Tian[1], Huaixuan Li[2,3], Chiho Yoon[2], Ryan A. Nelson[4], Ziling Li[1], Kenji Watanabe[5], Takashi Taniguchi[6], Dmitry Smirnov[7], Roland K. Kawakami[1], Joshua E. Goldberger[4], Fan Zhang[2], Chun Ning Lau[1]

[1] Department of Physics, The Ohio State University, Columbus, OH 43210.

[2] Department of Physics, The University of Texas at Dallas, 800 West Campbell Road, Richardson, Texas 75080-3021, USA

[3] Department of Physics, Carnegie Mellon University, Pittsburgh, Pennsylvania 15213, USA

[4] Department of Chemistry and Biochemistry, The Ohio State University, Columbus, OH 43210.

[5] Research Center for Electronic and Optical Materials, National Institute for Materials Science, 1-1 Namiki, Tsukuba 305-0044, Japan

[6] Research Center for Materials Nanoarchitectonics, National Institute for Materials Science, 1-1 Namiki, Tsukuba 305-0044, Japan

[7] National High Magnetic Field Laboratory, Tallahassee, FL 32310

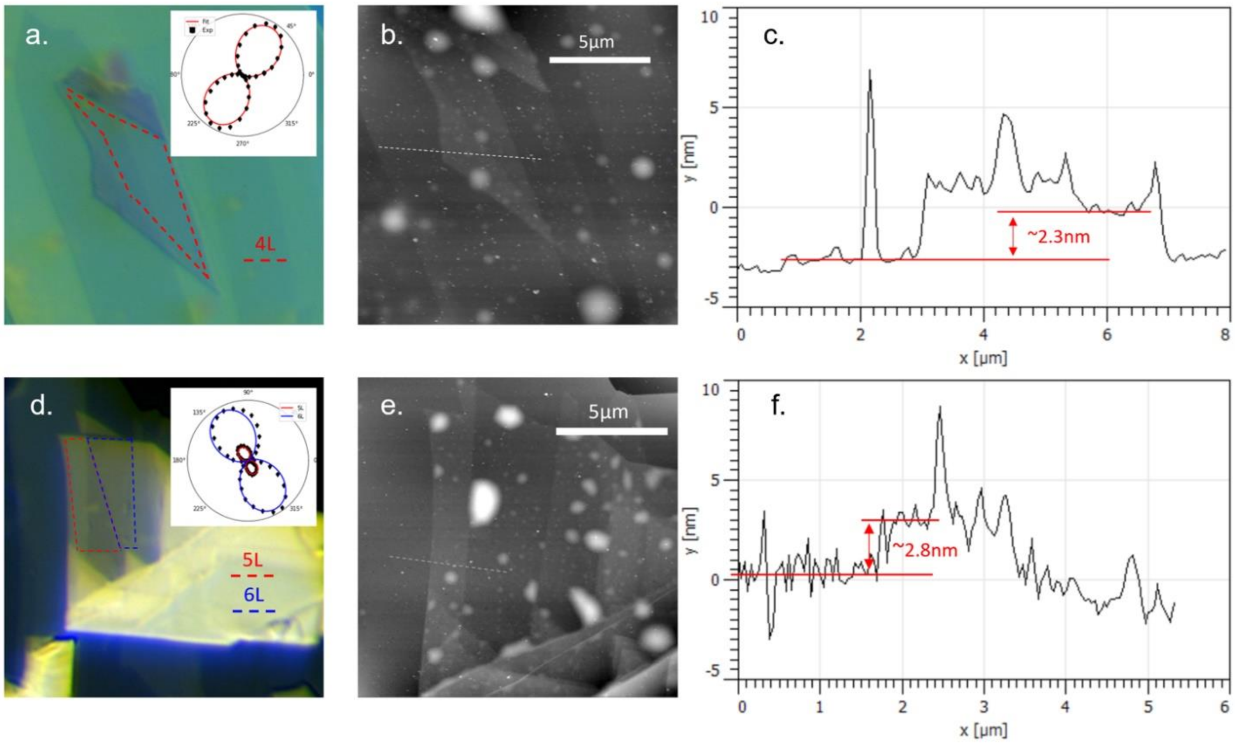

Supplementary Figure 1. Determine the Number of Layers of $PdSe_2$

(a). Optical images of a h-BN encapsulated 4-layer $PdSe_2$ flake with graphene contacts. Inserted: strong SHG signal on the 4L region, confirming the nature of an even-numbered layers. (b). AFM image of the 4-layer $PdSe_2$. (c). Line profile of the dotted line in (b), showing that the thickness of the 4-layer $PdSe_2$ is ~2.3 nm. (d). Optical images of a $PdSe_2$ flake with a monolayer step. Inserted: SHG intensity on 6L is ~4 times stronger than that on 5L. (e). AFM images of the 5L/6L $PdSe_2$. (f). Line profile of the dotted line in (e), showing that the thickness of the 5-layer $PdSe_2$ is ~2.8 nm.

Previous study[1] shows that the second harmonic generation (SHG) signals from even-numbered layers $PdSe_2$ are strong due to the broken inversion symmetry, while the odd-numbered layers $PdSe_2$ preserve inversion symmetry, therefore the SHG signals are negligible. To get an accurate estimation of the number of layers, we use Atomic Force Microscope (AFM) to measure the thickness of the encapsulated flake, meanwhile using the intensity of SHG signal to determine if the layer number is odd or even. Based on the thickness of several few layer $PdSe_2$, we determine the averaged monolayer thickness to be ~5.5 Å. The experimentally measured monolayer thickness is slightly higher than the theoretical number of ~ 4Å. We attribute the derivations to systematic errors related with the AFM resolution and increased uncertainty from h-BN encapsulation.

Supplementary Note 1

Polarization-resolved Second Harmonic Generation (SHG) Measurements

The SHG measurement used a Ti: sapphire laser for 800 nm excitation wavelength and ~150 fs pulses at a 76 MHz repetition rate. The laser beam passed through a linear polarizer, was reflected by a short pass dichroic beam splitter, and focused onto the sample using a 60x objective. The spot size was ~2 μm and the laser power at the sample was ~2 mW. The generated 400 nm SHG signal followed a reverse path through the objective and transmitted through the dichroic beam splitter. The SHG beam then went through an analyzer which was cross-polarized relative to the polarizer in the excitation line. A multimode fiber was used to collect the SHG emission and sent it to a spectrometer. To obtain the SHG intensity as a function of incident polarization angle, a broadband half waveplate was inserted in between the objective and the dichroic beam splitter. By rotating the half waveplate, the polarizations of both the 800 nm and 400 nm beams were rotated at the same time, which is equivalent to rotating the sample.

Supplementary Tabel 1: List of Devices

**Device**	**B1**	**A1**	**A2**	**A3**	**A4**	**A5**	**A6**
**Number of Layers**	**7L**	**5L**	**5L**	**7L**	**7L**	**3L**	**3L**

Supplementary Note 2

Effect of Few-Layer Graphene Contacts

We have ascertained that the observed oscillations are intrinsic properties of $PdSe_2$ without any contribution from the graphite, from the following observations:

First, the top gate covers only the $PdSe_2$ channel but not the graphene-contacted leads, thus it tunes only the carrier density in $PdSe_2$ without affecting that in the graphene contacts. Therefore, any response to V_{tg} arises only from $PdSe_2$. Meanwhile, since graphene contacts are on top of $PdSe_2$, the back gate also has limited tunability on the carrier density in graphene due to screening once $PdSe_2$ becomes conductive.

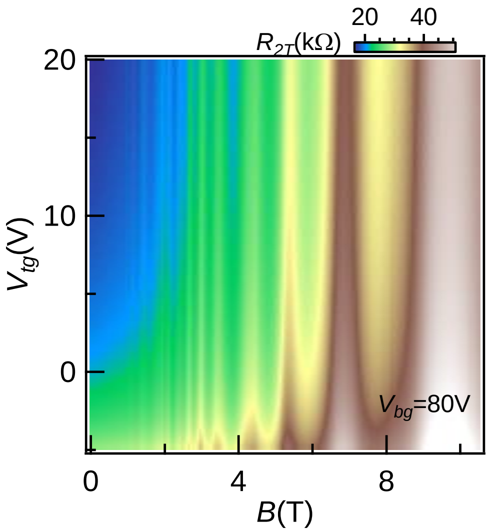

Supplementary Figure 2. Two-terminal resistance $R_{2T}(V_{tg},B)$ plot of a BN-encapsulated few layer $PdSe_2$ device(A6) with graphene contacts. The vertical features are periodic in $1/B$ and arises from quantum oscillations in the few-layer graphene

Second, all data presented in this paper are from four-probe measurements, which eliminate the contact resistance and therefore any contribution from the few-layer graphene contacts. In fact, in two- terminal measurements, few-layer graphene gives rise to a series of strong gate-independent oscillations that onset at small field (<1T) due to the high mobility nature of graphene, obscuring features from $PdSe_2$.

Third, at low fields, all quantum oscillations from $PdSe_2$ are 8-fold degenerate; in contrast, in the absence of broken symmetries, quantum oscillations in few-layer graphene are 4-fold degenerate, with the exception of the so-called zeroth-energy Landau level, which can be 4*L*-fold degenerate, arising from the orbital degeneracy of the lowest *L* Landau levels (here *L* is the number of layers) at the charge neutrality point.

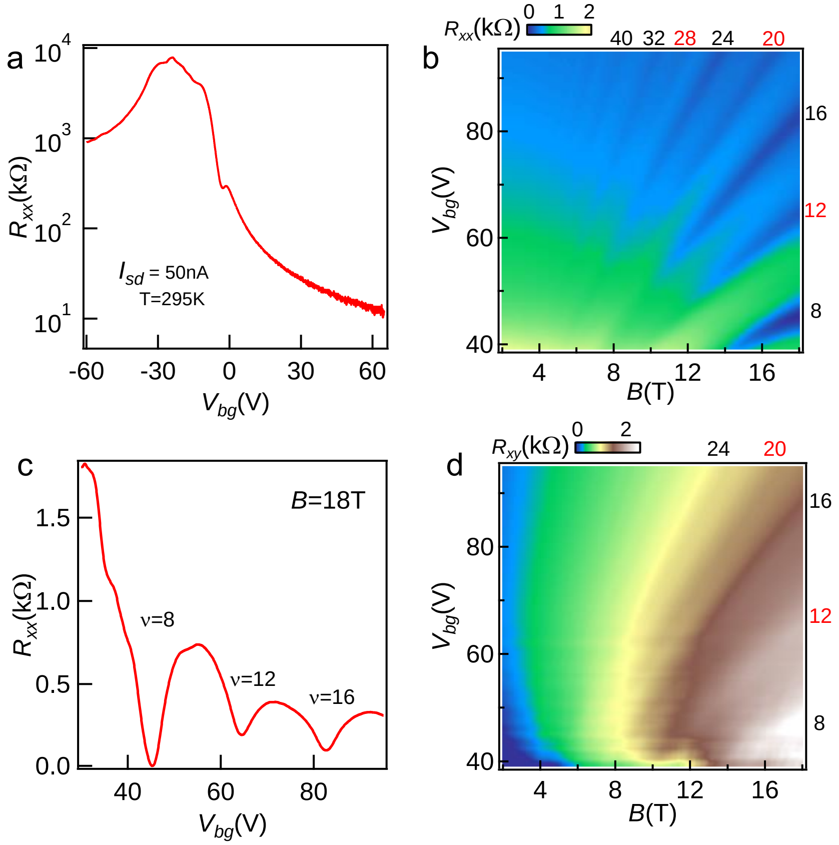

<table><tr><td>Supplementary Figure 3. Additional Transport Data (a) Field effect characteristic of device A2 at room temperature from -60V to +65V. (b). $R_{xx}(V_{bg}, B)$ of a 3-L device A5. (c) Line trace of $R_{xx}(V_{bg})$ at B=18T from (b). (d) $R_{xy}(V_{bg}, B)$ of A5. The numbers indicate the filling factors.</td></tr></table>

As shown in Supplementary Figure 3a, for hBN-encapsulated devices, the hole mobility is much lower than the electron mobility. For instance, R_{xx} for $V_{bg}<0$ is at least 2 orders of magnitudes higher than the optimized R_{xx} value at V_{bg}=60V, and hole mobility ~2 cm^2/Vs. Thus, no quantum oscillation is resolved from the valence band.

Supplementary Figure 3b shows the $R_{xx}(V_{bg}$, B) plot of a 3 layer device A5. At lower field, the Landau fan features resistance minima at filling factors $\nu=8N$, where N=1, 2, 3… is an integer denoting the Landau level (LL) index, as labeled by the black numbers. At higher field, the 8-fold degeneracy starting to be partially lifted, giving rise to minor R_{xx} dips between the major 8-fold R_{xx} minima at $\nu=4N_{odd}$, where N_{odd}= 3, 5, 7… is an odd integer, as labeled by the red numbers.

Supplementary Figure 3c shows a $R_{xx}(V_{bg})$ line trace at B=18T from Supplementary Figure 3b, the resistance at ν=8 reaches zero. We note that this is raw data, without any subtraction. Supplementary Figure 3d plots $R_{xy}(V_{bg}$, B) of A5, the numbers indicate the filling factors.

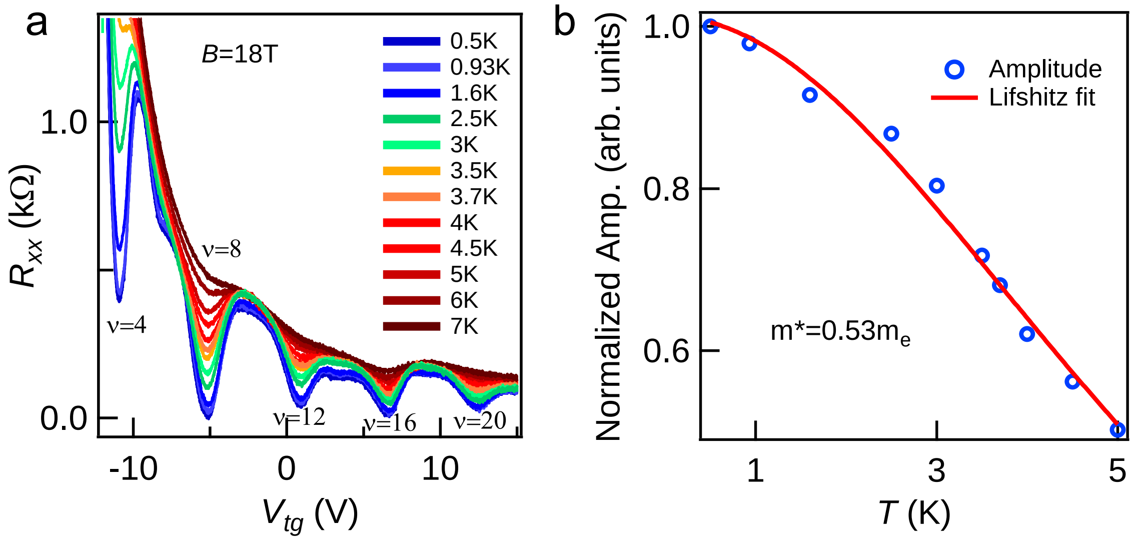

Supplementary Figure 4. Estimate of Effective mass at Higher Magnetic Field

(a). R_{xx} versus top gate voltages at B=18T and different temperatures. (b). Normalized amplitude of oscillations vs temperature. The red line is a fit to the Lifshitz-Kosevich equation.

Under a constant perpendicular field of B=10.5T, R_{xx} vesus V_{tg} was measured at different temperatures. The effective mass of the charge carriers is extracted by fitting the temperature dependence of the SdH oscillations amplitude to the Lifshitz-Kosevich (LK) formula, which yields an effective mass m*=0.37m_e, here m_e is the bare electron mass in vacuum. At an even larger field of B=18T, the effective mass extracted turns out to be m*=0.53m_e, almost a factor of two heavier than the effective mass estimated at B=7.5T. This enhancement may be due to one or both of the following factors. First, the LK formula is strictly applicable only in the limit of large N, where N is the number of occupied Landau levels; hence at higher field, the smaller N may lead to larger error bars in the extracted effective mass. Second, the enhanced electronic interactions at higher field may lead to increased effective mass at higher field when the 8-fold degeneracy starts to be lifting suggest enhanced interactions. Future experiments at high magnetic fields in the quantum Hall regime will be necessary to resolve this question.

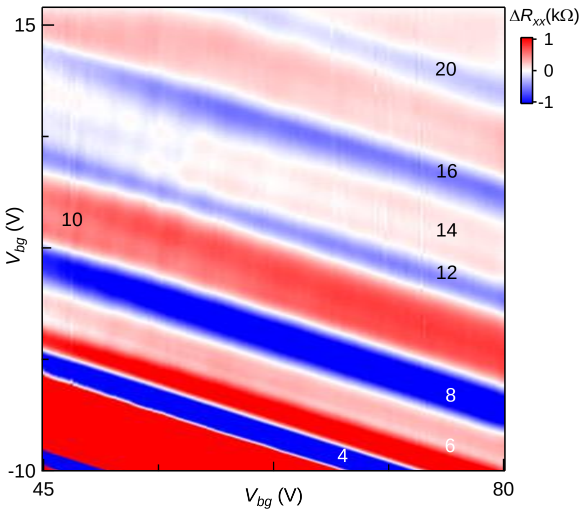

Supplementary Figure 5. Dependence on Out-of-Plane Displacement Field

The background-subtracted longitudinal resistance *R* as a function of voltages applied to back gate and top gate at *B*=18T. The numbers indicate the filling factor.

Supplementary Figure 5 plots the background-subtracted longitudinal resistance *R* from device A4 as a function of voltages applied to back gate and top gate at *B*=18T. The numbers indicate the filling factor ν. The device exhibits weak dependence on the out-of-plane displacement field, e.g. the quantum Hall states at ν =8 and 14 appears to be better resolved at larger displacement field, though further systematic studies will be necessary to ascertain the effect. Theoretically, the out-of-plane displacement field neither breaks the spatial symmetry that yields the 4-fold valley degeneracy nor the time-reversal symmetry that dictates the 2-fold spin degeneracy; thus, the effect of the displacement field may be relatively subtle.

Supplementary Note 3

First-principles calculations of electronic band structures

Our first-principles calculations based on density functional theory were performed using the Vienna *ab initio* simulation package (VASP) [2, 3] with the projected augmented-wave method [4] and exchange-correlation of the Perdew-Burke-Erbzerhof type within the generalized gradient approximation (GGA) [5]. The DFT-D3 functional was used to describe the van der Waals interaction[6]. A vacuum buffer space of 2 nm was applied to avoid unnecessary interactions along *z*-axis between adjacent slabs. The plane-wave energy cutoff was set to be 400 eV. A *k*-point grid of 5×5×1 for the first Brillouin zone was used in all our self-consistent calculations. To obtain the equal-energy contours of bands, a 16×16×1 grid was used for the first-principles calculations for the rectangular region with its four vertices being the M point, the middle point of MX, the middle point of MΓ, and the middle point of MY; this region is 1/16 of the first Brillouin zone. The MATLAB function interp2 was then used to interpolate the first-principle results and generate the equal-energy contours at specific energies.

Supplementary Figure 6 plots the bulk and 1-7 layer $PdSe_2$ band structures without (left) and with (right) spin-orbit couplings (SOC). Evidently, for thicker layers and lower conduction bands, the SOC effects are negligibly weak. For this reason, as shown in Supplementary Figure 7, the equal-energy contours for the lowest conduction band of 7-layer $PdSe_2$ without (left) and with SOC (right) have no noticeable difference.

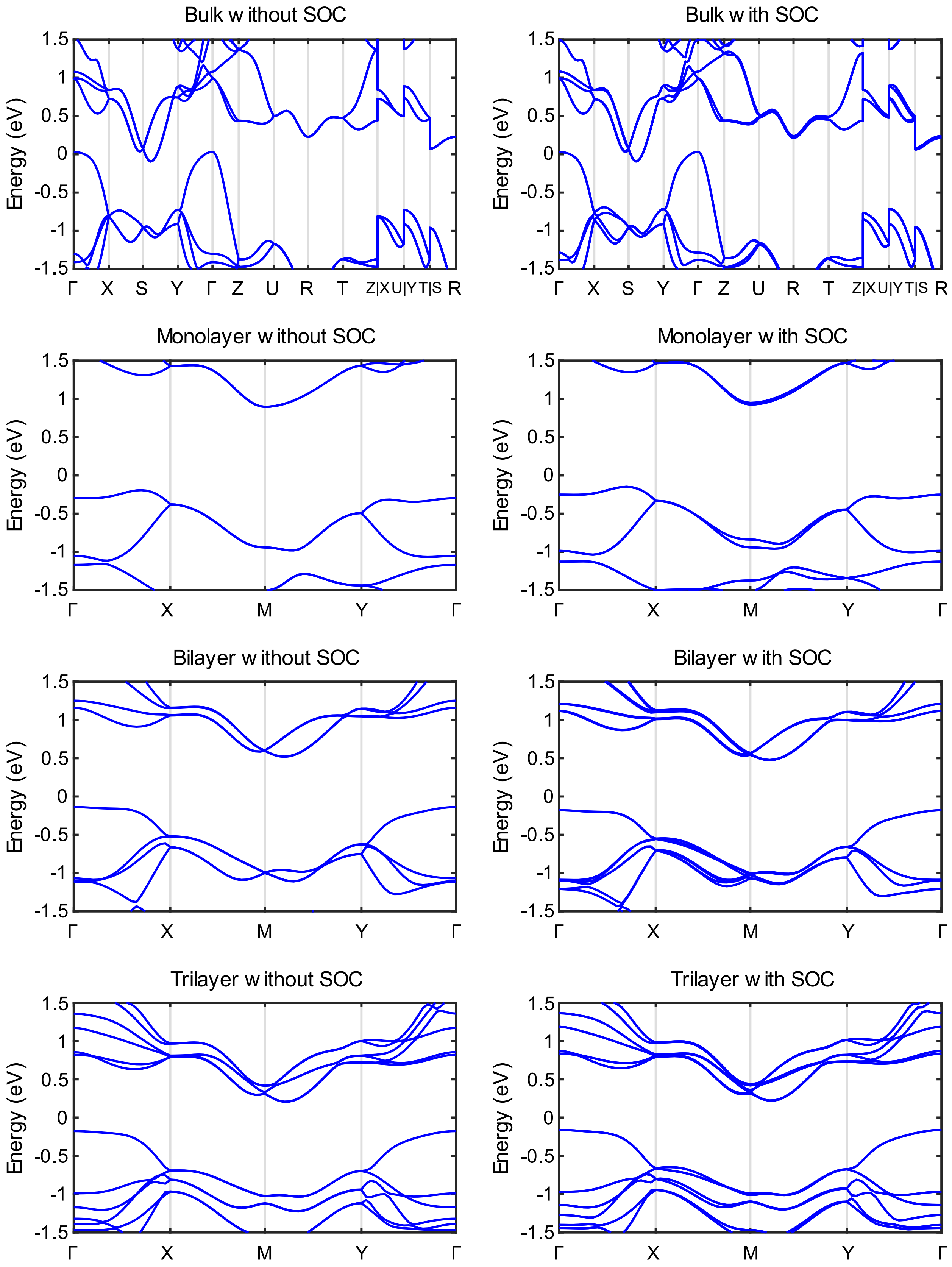

Bulk w ithout SOC
Bulk w ith SOC
Monolayer w ithout SOC
Monolayer w ith SOC
Bilayer w ithout SOC
Bilayer w ith SOC
Trilayer w ithout SOC
Trilayer w ith SOC
Energy (eV)
1.5
1
0.5
0
-0.5
-1
-1.5
Γ X S Y Γ Z U R T Z|X U|Y T|S R
Γ X M Y Γ

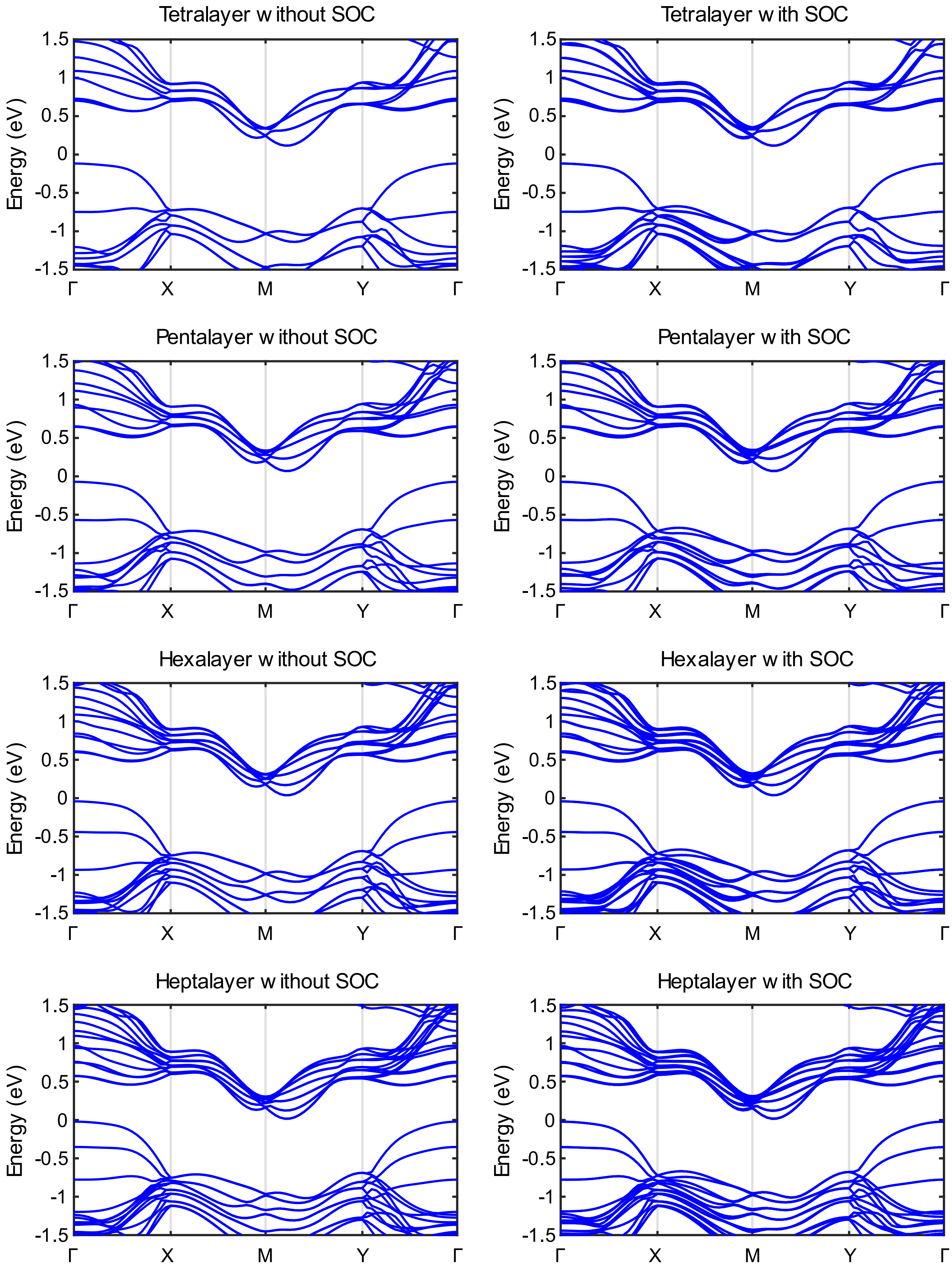

Supplementary Figure 6. Bulk and 1-7 layers $PdSe_2$ band structures without (left) and with SOC (right).

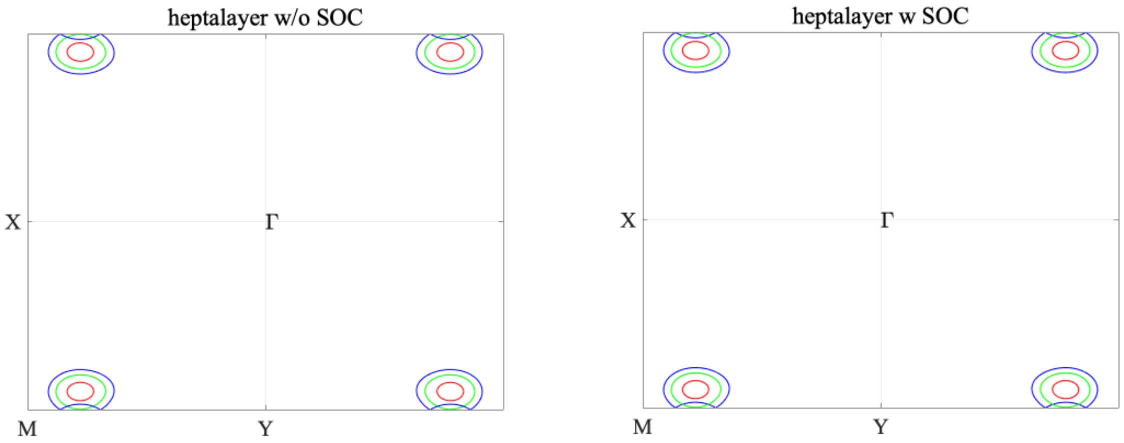

Supplementary Figure 7. Equal-energy contours for the lowest conduction band of 7-layer PdSe2 without (left) and with SOC (right). The two cases have no noticeable difference. The corresponding energies are 10, 35, and 60 meV above the lowest conduction band minimum.

Supplementary References

[1] J. Yu, X. Kuang, J. Li, J. Zhong, C. Zeng, L. Cao, Z. Liu, Z. Zeng, Z. Luo, T. He, A. Pan, and Y. Liu, Giant nonlinear optical activity in two-dimensional palladium diselenide, *Nat. Commun.* 12, 1083 (2021).

[2] G. Kresse, and J. Hafner, Ab initio molecular dynamics for liquid metals, *Phys. Rev. B* 47, 558 (1993).

[3] G. Kresse, and J. Furthmüller, Efficient iterative schemes for ab initio total-energy calculations using a plane-wave basis set, *Phys. Rev. B* 54, 11169 (1996).

[4] P. E. Blöchl, Projector augmented-wave method, *Phys. Rev. B* 50, 17953 (1994).

[5] J. P. Perdew, K. Burke, and M. Ernzerhof, Generalized Gradient Approximation Made Simple, *Phys. Rev. Lett.* 77, 3865 (1996).

[6] S. Grimme, J. Antony, S. Ehrlich, and H. Krieg, A consistent and accurate *ab initio* parametrization of density functional dispersion correction (DFT-D) for the 94 elements H-Pu, *The Journal of Chemical Physics* 132, 154104 (2010).
